# Identification of biomarkers in multiple myeloma: A comprehensive study combining microarray analysis and Mendelian randomization

**DOI:** 10.1111/jcmm.18504

**Published:** 2024-06-26

**Authors:** Yidong Zhu, Jun Liu, Bo Wang

**Affiliations:** ^1^ Department of Traditional Chinese Medicine, Shanghai Tenth People's Hospital, School of Medicine Tongji University Shanghai China; ^2^ Department of Endocrinology, Yangpu Hospital, School of Medicine Tongji University Shanghai China

**Keywords:** biomarker, Mendelian randomization, multiple myeloma, myeloperoxidase, tumour immunity

## Abstract

Despite remarkable advancements in the treatment of multiple myeloma (MM), relapse remains a challenge. However, the mechanisms underlying this disease remain unclear. This study aimed to identify potential biomarkers that could open new avenues for MM treatment. Microarray data and clinical characteristics of patients with MM were obtained from the Gene Expression Omnibus database. Differential expression analysis and protein–protein interaction (PPI) network construction were used to identify hub genes associated with MM. Predictive performance was further assessed using receiver operating characteristic curves and nomogram construction. Functional enrichment analysis was conducted to investigate possible mechanisms. Mendelian randomization (MR) was used to evaluate the causal relationship between the crucial gene and MM risk. Topological analysis of the PPI network revealed five hub genes associated with MM, with myeloperoxidase (*MPO*) being the key gene owing to its highest degree and area under the curve values. *MPO* showed significant differences between patients with MM and controls across all datasets. Functional enrichment analysis revealed a strong association between *MPO* and immune‐related pathways in MM. MR analysis confirmed a causal relationship between *MPO* and the risk of MM. By integrating microarray analysis and MR, we successfully identified and validated *MPO* as a promising biomarker for MM that is potentially implicated in MM pathogenesis and progression through immune‐related pathways.

## 1 INTRODUCTION

Multiple myeloma (MM) is a malignant neoplasm characterized by the accumulation of plasma cells in the bone marrow, resulting in renal failure, hypercalcemia, bone destruction and anaemia due to marrow failure.^1^ In the United States, MM accounts for approximately 1.8% of all cancers and 19.1% of hematologic malignancies.^2^ The median age at MM diagnosis was 69, with the majority of cases occurring in individuals aged 65–74.^3^ Over the past two decades, significant progress has been made in the development of novel therapies for MM, including proteasome inhibitors, immunomodulatory drugs, monoclonal antibodies and CAR T‐cell therapies, resulting in improved patient outcomes.^4^, ^5^, ^6^, ^7^, ^8^, ^9^ Despite these advancements, the survival prognosis in clinical practice remains poor, presenting a significant challenge attributed to drug resistance and frequent recurrence.^10^, ^11^ Numerous signalling pathways, such as Ras/Raf/MEK/MAPK, JAK/STAT, NF‐kB and Wnt/β‐catenin, have been implicated in MM development through oncogenic mutations in pivotal genes.^12^, ^13^, ^14^ Recent studies have emphasized the substantial role of the bone marrow (BM) microenvironment in MM development and progression.^15^, ^16^, ^17^ However, the underlying mechanism remains unclear. Given the evident heterogeneity in the pathogenesis, clinical presentation and prognosis of patients with MM, there is considerable research potential to identify more effective and dependable molecular markers for personalised treatment strategies.

In the era of big biological data, the analysis of vast datasets is feasible by integrating biology, computer science and information technology.^18^, ^19^ High‐throughput microarray technology is widely used for the molecular diagnosis, classification and prognosis of MM, providing a novel avenue for the identification of potential biomarkers and pathways.^20^, ^21^, ^22^, ^23^, ^24^, ^25^ Despite the significant roles of certain biomarkers in the onset and progression of the disease during subsequent validation, challenges persist in establishing causality owing to issues such as reverse causation and confounding factors. Mendelian randomization (MR), an analytical approach aligned with Mendel's law of inheritance, utilizes single nucleotide polymorphisms (SNPs) as instrumental variables (IVs) to infer causality between a modifiable exposure and a clinically relevant outcome, which could address these limitations.^26^, ^27^ The underlying principle of IV analysis in MR relies on the random assortment of genetic variants during meiosis.^28^ In this regard, the MR framework parallels that of a randomised controlled trial because the random assortment of genetic variants mimics the random allocation of participants to different treatment arms.^29^ Genetic variants are established at conception and remain unaffected by the development of outcomes or external (i.e., environmental) factors; therefore, they are generally considered independent of confounding factors and reverse causation.^30^ Furthermore, in the modern genomic era, genotypes are less susceptible to measurement errors, thereby minimizing potential bias.^31^ Accumulating evidence substantiates the value of MR as an effective approach for identifying risk factors for MM.^32^, ^33^, ^34^, ^35^, ^36^ However, the specific application of combining microarray analysis with the MR approach to identify MM biomarkers remains unexplored.

Here, we conducted a differential expression analysis of MM and control samples using microarray data from the Gene Expression Omnibus (GEO) database. Subsequently, topological analysis based on a protein–protein interaction (PPI) network was used to identify hub genes associated with MM. Predictive performance was further assessed using receiver operating characteristic (ROC) curves and nomogram construction. Functional enrichment analysis was performed to investigate the underlying mechanisms. The validity of the crucial gene was confirmed by external datasets. Furthermore, MR was used to examine the causal relationship between the gene level and MM risk. Our findings revealed dependable biomarkers associated with MM and shed light on the underlying molecular mechanisms that have implications for facilitating the early detection of MM in clinical practice and identifying potential targets for future drug development.

## 2 MATERIALS AND METHODS

### 2.1 Data acquisition

We obtained four datasets from the GEO database (https://www.ncbi.nlm.nih.gov/geo/) consisting of MM and control samples: GSE6477, GSE24870, GSE118985 and GSE125361. Table S1 provides a comprehensive overview of the datasets used in the study. To eliminate batch effects, normalisation was applied to the raw data of all the analysed datasets. The GSE118985 dataset was used as the training cohort for subsequent analyses, and the GSE6477, GSE24870 and GSE125361 datasets were used as diverse external validation cohorts to confirm the findings.

### 2.2 Identification of differentially expressed genes (DEGs)

Differential expression analysis of MM and control samples was performed using the ‘LIMMA’ package. To identify DEGs, a criterion of |log(fold change)| >1 and adjusted *p* < 0.05 was applied. The visualization of DEGs was achieved through the utilization of the ‘pheatmap’ and ‘ggplot2’ packages, which generated a heatmap and volcano plot, respectively.

### 2.3 Construction of a PPI network

A PPI network was created using the STRING (http://string‐db.org) database to identify the interactions among the identified DEGs. Subsequently, the constructed network was imported into Cytoscape software (version 3.8.2). Topological analysis was performed using the CytoHubba tool to rank significant genes within the PPI network. The five genes with the highest degree values within the PPI network were identified as hub genes.

### 2.4 Nomogram construction and ROC analysis

A nomogram was developed to predict disease risk using hub genes. The performance of the nomogram model was assessed using the calibration curve. Moreover, ROC curves were generated to assess the accuracy and specificity of these genes for diagnosing MM. The analysis and visualisation of these assessments were conducted using the ‘rms’, ‘rmda’, ‘glmnet’ and ‘pROC’ packages.

### 2.5 Gene validation

Based on the degree and area under the curve (AUC) values of the identified hub genes, myeloperoxidase (*MPO*) was chosen as the most critical gene for subsequent analyses. To confirm the diagnostic value of *MPO*, differential expression analysis was conducted using diverse validation cohorts (GSE6477, GSE24870 and GSE125361). The findings from these analyses were visualized using the ‘ggpubr’ package.

### 2.6 Functional enrichment analysis

To explore potential mechanisms, DEGs were subjected to Gene Ontology (GO) and Kyoto Encyclopedia of Genes and Genomes (KEGG) analyses. The CIBERSORT algorithm was used to compare the levels of 22 distinct infiltrating immune cell types between patients with MM and controls. Spearman's correlation analysis was performed to assess the relationship between *MPO* and infiltrating immune cells. These analyses were performed, and their results were visualized using various packages, including ‘clusterProfiler’, ‘enrichplot’, ‘org.Hs.eg.db’, ‘pheatmap’, ‘vioplot’, ‘reshape2’ and ‘ggplot2’.

### 2.7 MR analysis

Two‐sample MR was used to investigate the causal association between the critical gene and MM risk. *MPO*, identified as the critical gene, and MM, specified as the disease of interest, were used for the MR analysis. To mitigate the potential impact of population stratification, we focused exclusively on European populations in our primary analyses. A summary of genetic association data for *MPO* (ID: prot‐b‐29) and MM (ID: ieu‐b‐4957) was obtained from publicly available Genome‐Wide Association Studies datasets (GWAS, https://gwas.mrcieu.ac.uk). After harmonizing the effect alleles across *MPO* and MM, several MR approaches were applied to estimate the causal effect of *MPO* on MM, including the inverse variance weighted (IVW) method, MR‐Egger regression, weighted median approach, simple mode and weighted mode. Among these methods, if all included SNPs satisfied the assumption of being valid instruments in this analysis, the IVW method would be capable of providing accurate estimates of the causal effect between exposure and outcome and would be considered the primary approach.^37^ Sensitivity analysis plays a pivotal role in detecting potential horizontal pleiotropy and heterogeneity in MR studies. Heterogeneity markers (Cochran's Q‐derived *p* < 0.05) were used to represent potential horizontal pleiotropy. Additionally, leave‐one‐out analysis was conducted to assess whether any single SNP drove or biased the MR estimates. The ‘TwoSampleMR’ package was employed for conducting the MR analysis.

### 2.8 Statistical analysis

Data was analysed using R software (version 4.3.0). For quantitative variables, differences between groups with normally distributed variables were analysed using the Student's *t*‐test, while the Wilcoxon test was employed for skewed data. Spearman's correlation analysis was used to assess the relationships among the variables. A two‐sided *p*‐value of less than 0.05 was considered indicative of a statistically significant difference.

## 3 RESULTS

### 3.1 Identification of DEGs

**FIGURE 1:**
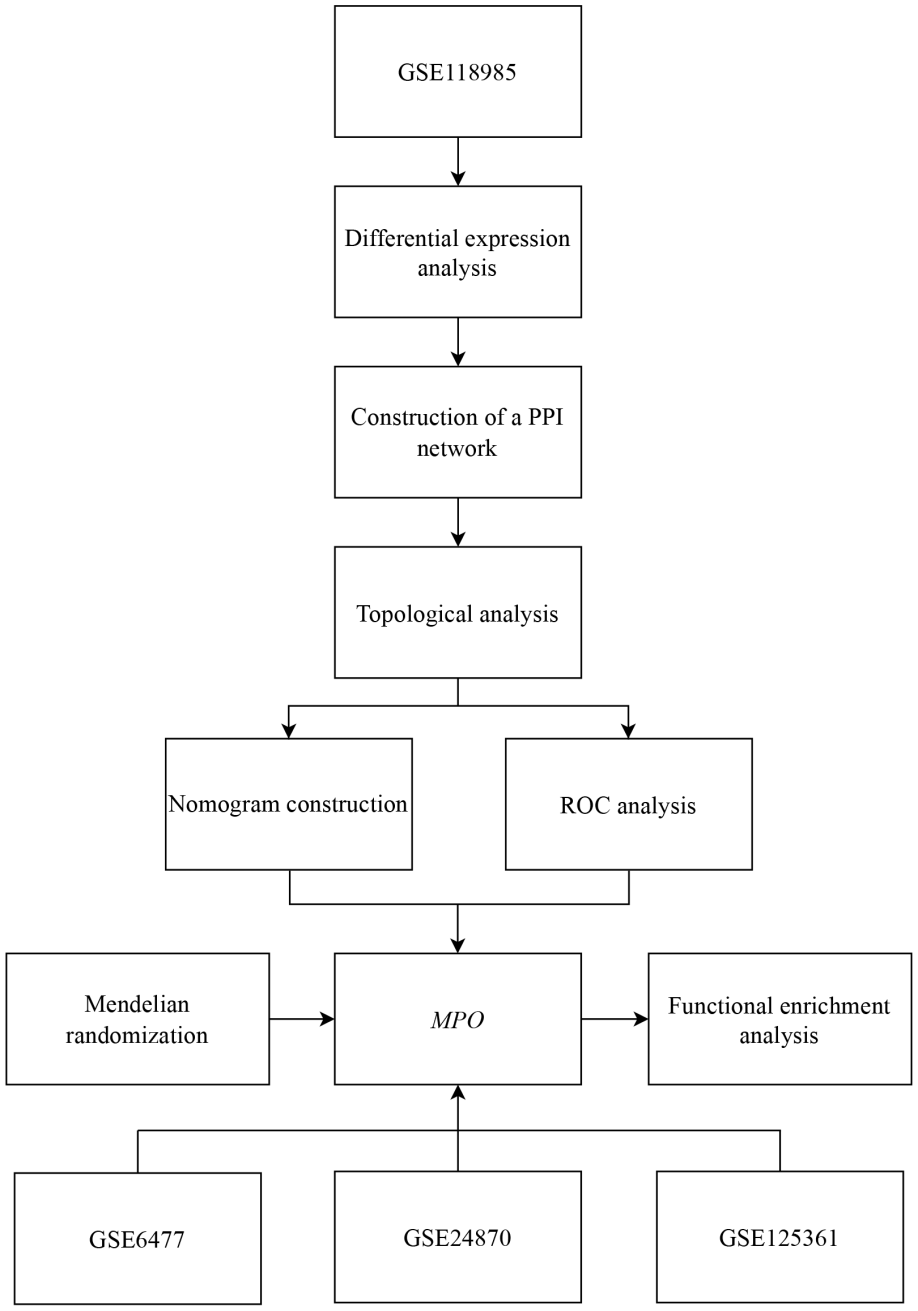
Flowchart of this study. PPI: protein–protein interaction; MM, multiple myeloma; ROC, receiver operating characteristic.

**FIGURE 2:**
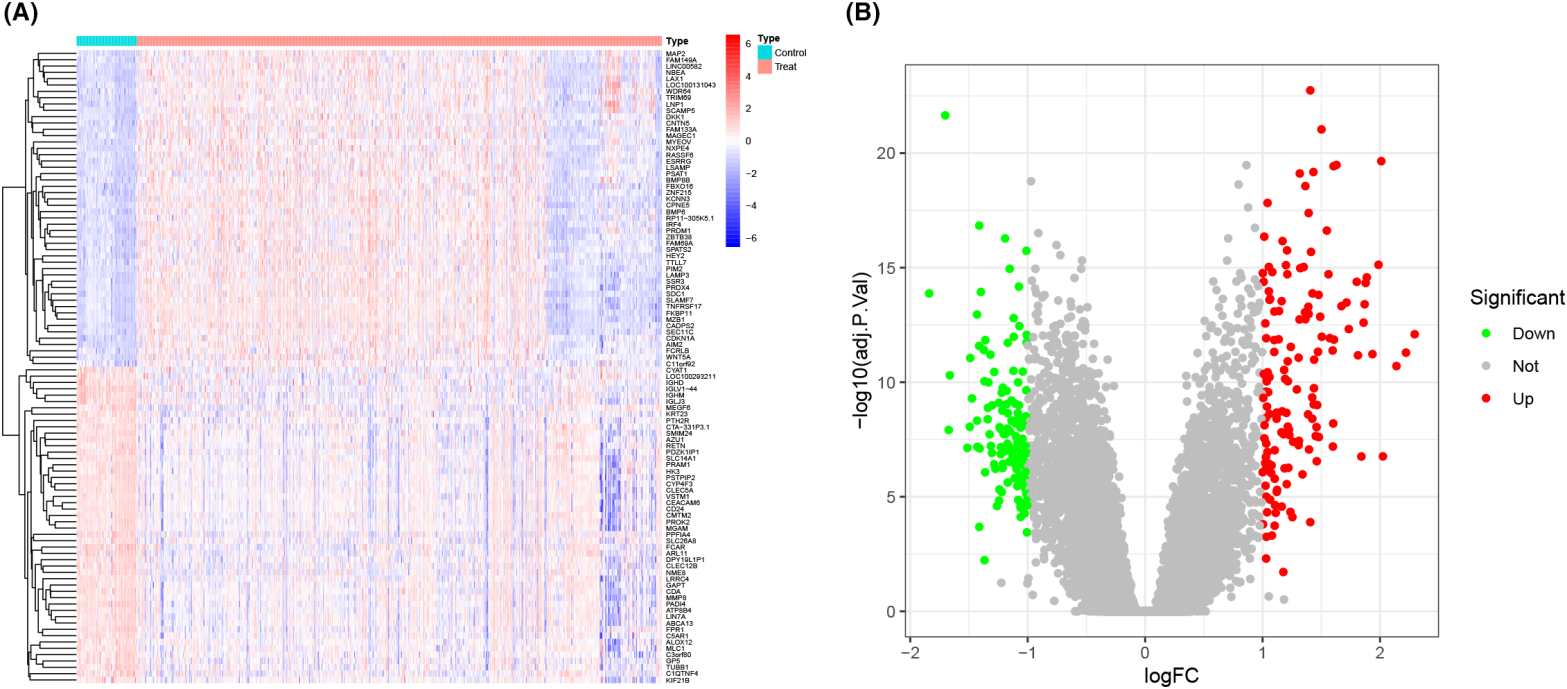
Identification of DEGs between MM and control samples. (A) Heatmap of DEGs. (B) Volcano plot of DEGs. DEGs, differentially expressed genes; MM, multiple myeloma.

Figure 1 shows a flowchart of the analysis conducted in this study. By applying predefined criteria to the training set, we identified 269 DEGs between the MM and control samples. Among these DEGs, 145 were upregulated and 124 were downregulated specifically in MM (Figure 2A,B).

### 3.2 Construction of a PPI network

**FIGURE 3:**
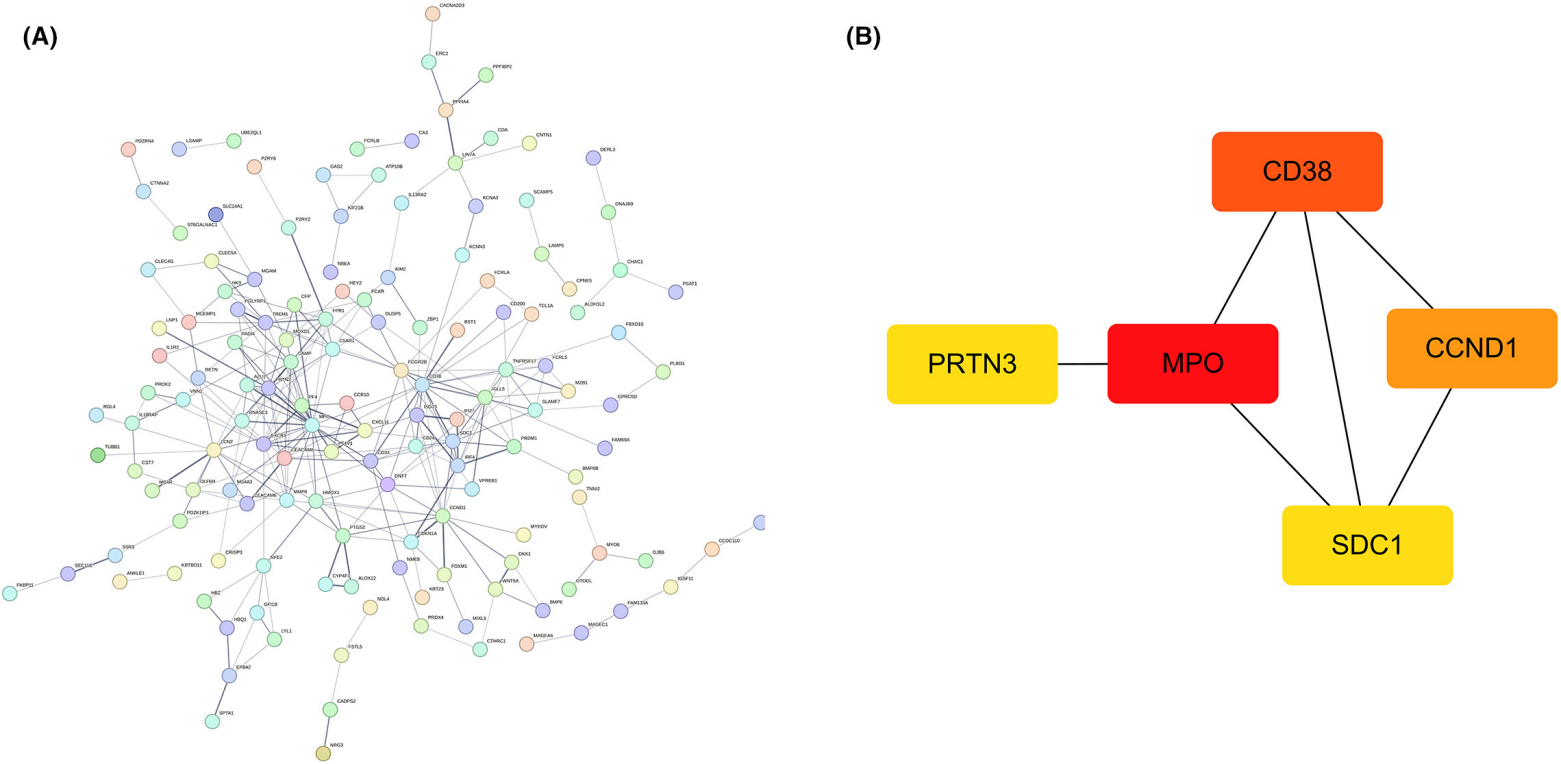
Construction of a PPI network. (A) PPI network of DEGs. (B) Top five genes with the highest degree values. DEGs, differentially expressed genes; PPI, protein–protein interaction.

A PPI network of DEGs was constructed using the STRING database to elucidate intergenic interactions (Figure 3A). Next, using topological analysis with the CytoHubba tool, we identified the five genes with the highest degree values as hub genes: *MPO*, *CD38*, *CCND1*, *LCN2* and *SDC1* (Figure 3B).

### 3.3 Nomogram construction and ROC analysis

**FIGURE 4:**
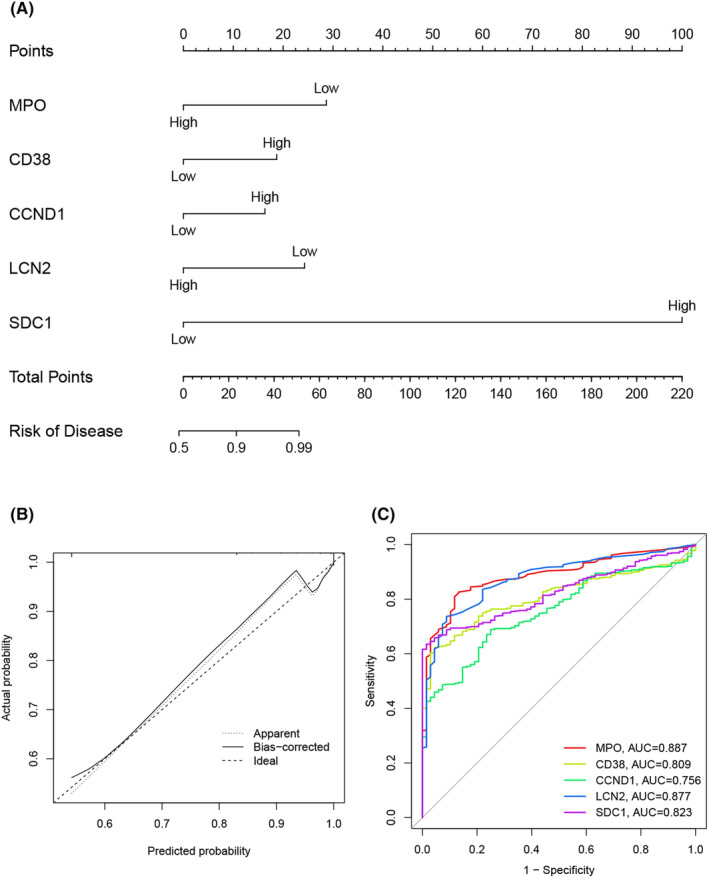
Nomogram Construction. (A) MM risk prediction nomogram, (B) calibration curve and (C) ROC curves of hub genes. MM, multiple myeloma; ROC, receiver operating characteristic.

We developed a nomogram incorporating hub genes to predict MM risk (Figure 4A). The calibration curve showed excellent agreement between the observed outcomes and the predicted risk probabilities (Figure 4B). ROC curves were subsequently calculated for the hub genes to evaluate their diagnostic performance for MM. The AUC values for *MPO*, *CD38*, *CCND1*, *LCN2* and *SDC1* were 0.887, 0.809, 0.756, 0.877 and 0.823, respectively (Figure 4C). *MPO* exhibited the highest degree and AUC values among the hub genes, indicating that it was the most crucial gene for further analysis.

### 3.4 Gene validation

**FIGURE 5:**
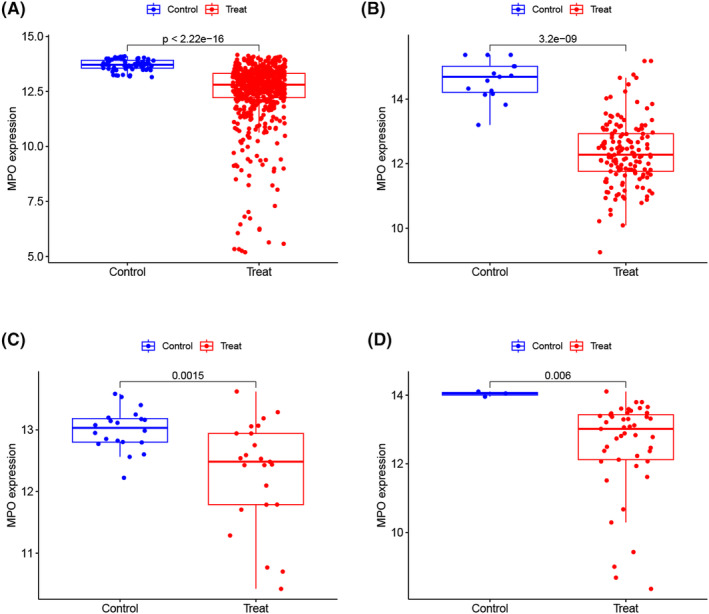
Gene validation. *MPO* in the training cohort (A), GSE6477 dataset (B), GSE24870 dataset (C) and GSE125361 dataset (D).

To validate the significance of the focal gene *MPO*, we assessed its expression in three external datasets comprising samples from patients with MM and controls. In the training cohort, the expression pattern of *MPO* showed a significant difference in patients with MM (*p* < 2.22e−16) in comparison to controls (Figure 5A), and this trend remained consistent in all the external datasets. Compared to that in controls, the expression levels of *MPO* exhibited a significant decrease in patients with MM across the GSE6477 dataset (*p* = 3.2e−09), the GSE24870 dataset (*p* = 0.0015) and the GSE125361 dataset (*p* = 0.006; Figure 5B–D).

### 3.5 Functional enrichment analysis

**FIGURE 6:**
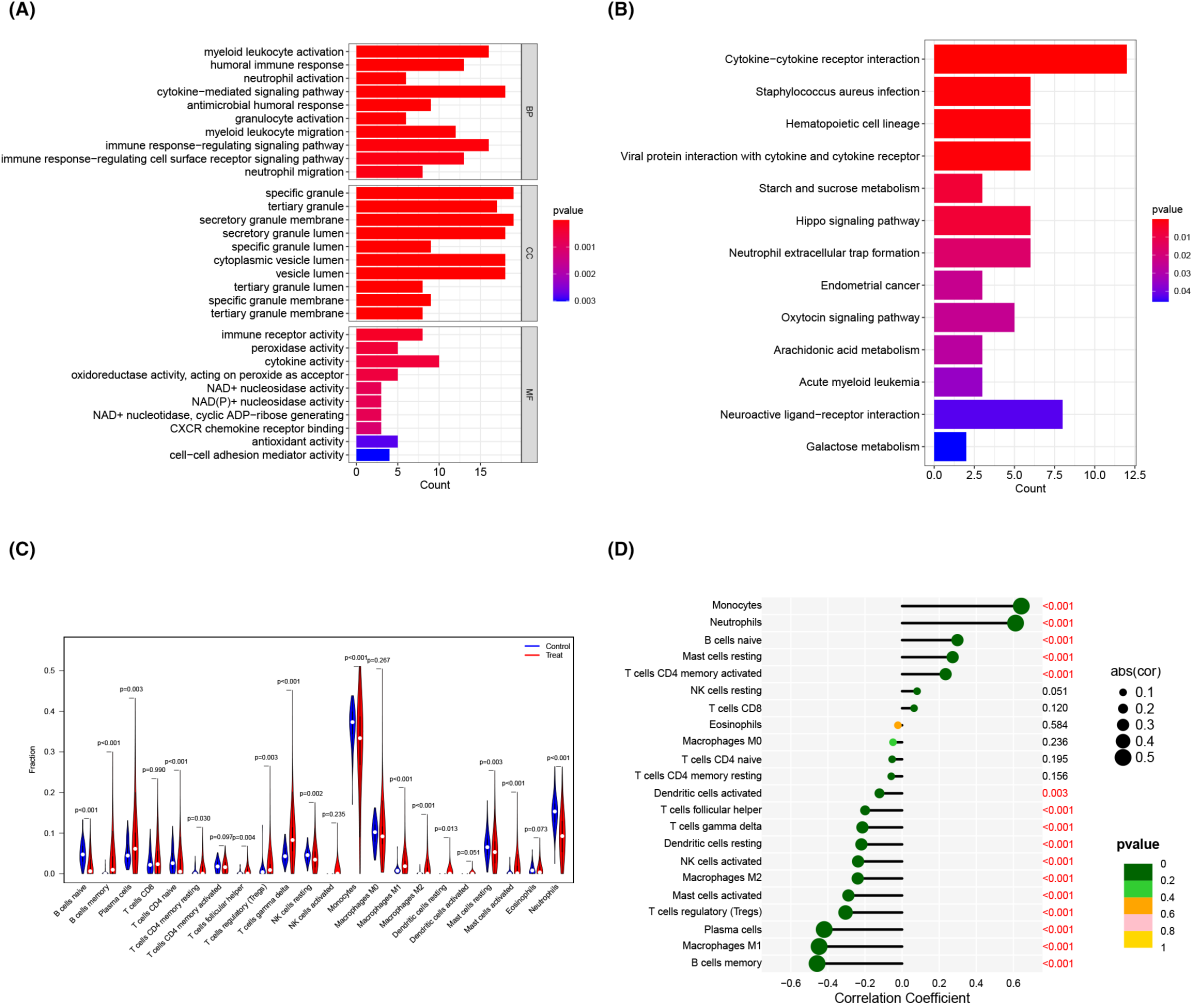
Functional enrichment analysis. (A) The bar plot of Gene Ontology (GO) analysis; (B) The bar plot of Kyoto Encyclopedia of Genes and Genomes (KEGG) analysis; (C) The vioplot of immune‐related cells; (D) The lollipop chart of correlations between *MPO* and immune‐related cells.

GO functional analysis revealed that the biological processes primarily encompassed myeloid leukocyte activation, humoral immune response, neutrophil activation and cytokine‐mediated signalling pathway (Figure 6A). Additionally, KEGG analysis indicated the prominent involvement of cytokine–cytokine receptor interaction, *Staphylococcus aureus* infection, haematopoietic cell lineage and viral protein interaction with cytokine and cytokine receptor (Figure 6B). Subsequently, we compared the enrichment levels of infiltrating immune cells between MM and control samples. The results revealed significant differences in 16 of 22 infiltrating immune cell types, highlighting the potential role of immunological dysfunction in the pathogenesis and progression of MM (Figure 6C). Furthermore, correlation analysis established a significant association between *MPO* and infiltrating immune cells (Figure 6D), suggesting that *MPO* may exert a profound influence on the immunological infiltration status of patients with MM.

### 3.6 MR

**FIGURE 7:**
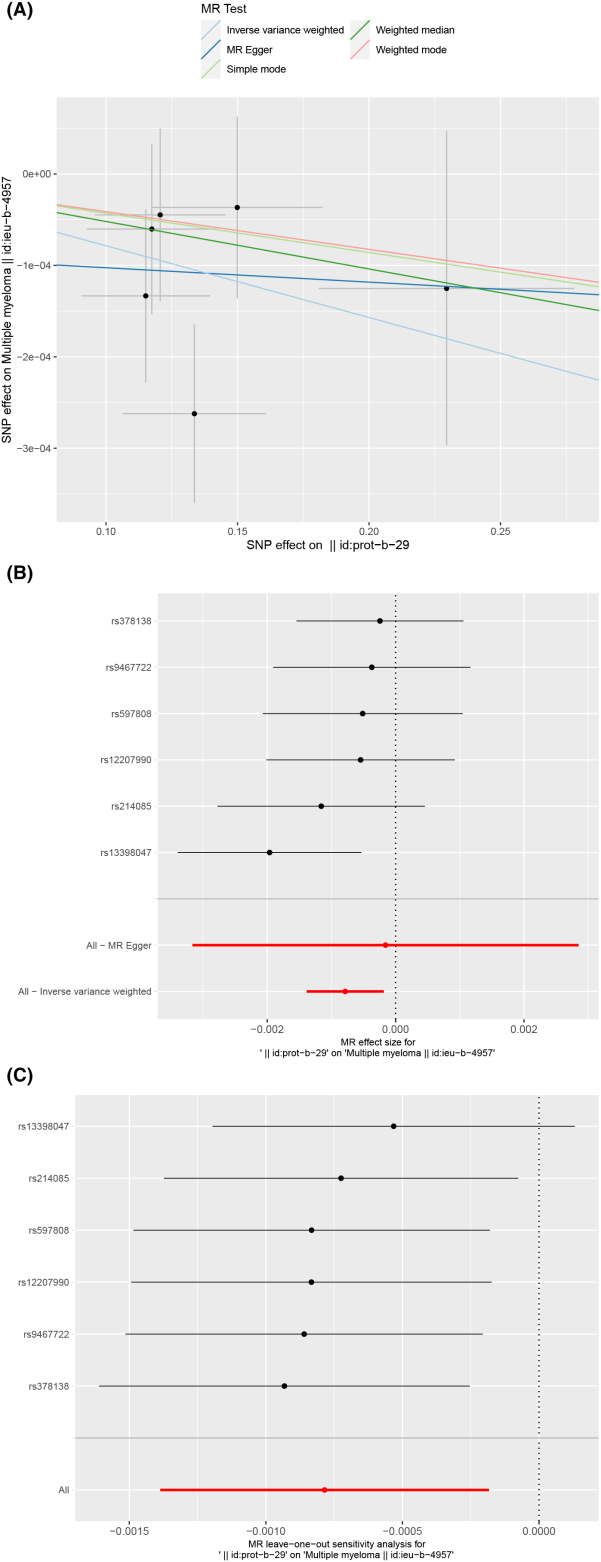
Mendelian randomization. (A) Scatter plot illustrating the causal effect of *MPO* on multiple myeloma (MM) risk. The horizontal axis represents the impact of single nucleotide polymorphisms (SNPs) on the exposure factor, whereas the vertical axis represents their impact on the outcome. Each black dot corresponds to an analysed SNP. (B) Forest plot showing the causal effect of each SNP on MM risk. (C) A leave‐one‐out plot was used to visualize the causal effect of *MPO* on MM risk, while excluding one SNP at a time.

The SNP characteristics of *MPO* and MM are shown in Table S2. Our analysis confirmed that all SNPs served as strong instrumental variables. Using the IVW method, we observed a significant association between *MPO* levels and the risk of MM (odds ratio [OR] = 0.9992, 95% confidence interval [CI] = 0.9986–0.9998, *p* = 0.0106). The other methods yielded similar risk estimates without statistical significance (Table S3 and Figure 7A,B). Nonetheless, the consistent trends in the ORs from these methods indicated that increased levels of the exposure factor corresponded to a decreased risk of developing the disease. Our MR analysis indicated no evidence of heterogeneity or horizontal pleiotropy, as shown in Table S4 and Table S5. Moreover, we systematically recalculated the MR analysis by sequentially excluding each SNP (Figure 7C). The results remained stable, indicating that the inclusion of all the SNPs yielded significant causal relationships. This further underscores the absence of a dominant SNP in *MPO* levels and MM, thus confirming the validity of our previous MR outcomes.

## 4 DISCUSSION

The emergence of novel treatment strategies has improved survival outcomes for patients with MM. Nevertheless, the survival prognosis in clinical practice remains poor due to drug resistance and frequent recurrence.^38^ The primary achievement of this study was the successful identification of MM biomarkers using microarray analysis and MR. Functional enrichment analysis revealed a significant correlation between immune‐related pathways and MM. Furthermore, MR analysis confirmed a causal association between *MPO* levels and the risk of MM. Overall, the integration of microarray analysis and MR has yielded valuable evidence for identifying biomarkers and elucidating the molecular mechanisms underlying the disease. This discovery offers potential targets for diagnosis and treatment and contributes to the advancement of medical research in this field.

In this study, we performed differential expression analysis of microarray datasets to identify 269 DEGs that distinguished MM samples from control samples. Subsequently, topological analysis of the PPI network based on the DEGs allowed us to select five genes with the highest degree values as hub genes: *MPO*, *CD38*, *CCND1*, *LCN2* and *SDC1*. Among these genes, *MPO* exhibited the highest degree and AUC values, establishing it as a focal gene for further investigation. The AUC value for *MPO* was 0.887, indicating excellent predictive performance in the diagnosis of MM. Furthermore, consistent with the training cohort and various validation cohorts, the expression patterns of *MPO* were significantly downregulated in patients with MM compared to those in control samples. *MPO* is a heme protein synthesized during myeloid differentiation and is a major component of neutrophil azurophilic granules.^39^ *MPO* plays a crucial role in microbicidal activity against a wide range of organisms by catalysing the formation of reactive oxygen intermediates.^40^, ^41^ In addition to its antimicrobial function, *MPO* acts as a local mediator of inflammation and is an important therapeutic target for inflammatory diseases. The deficiency of *MPO* leads to an exaggerated inflammatory response and affects various neutrophil functions, including cytokine production.^42^ Recently, there has been growing interest in studying neutrophils within a diverse range of innate immune cells present in the tumour microenvironment.^43^, ^44^ Neutrophil‐derived *MPO* has been implicated in cancer development and progression.^45^ High tumour infiltration by *MPO*‐expressing cells in breast and colorectal cancers has been associated with a significant improvement in prognosis.^46^, ^47^ Additionally, low levels of preoperative serum *MPO* may identify patients at high risk of recurrence and death following resection of colorectal liver metastases.^48^ However, there is limited research on the precise role of *MPO* in patients with MM.

Functional enrichment analysis was performed to investigate the underlying mechanisms. GO and KEGG analyses identified a significant enrichment of immune‐related pathways and diseases. Immune cell analysis revealed significant disparities in 16 of the 22 infiltrating immune cells, highlighting a strong association between MM and immunodeficiency. Extensive evidence suggests that myeloma downregulates antigens recognized by cellular immunity and modulates the BM microenvironment to facilitate uncontrolled tumour proliferation and apoptotic resistance, thereby impeding antitumour immunity.^49^, ^50^, ^51^ This phenomenon is characterized by disrupted immune surveillance, diminished antibody production, dysregulated T and NK cell populations, impaired antigen presentation, elevated expression of inhibitory surface ligands and the recruitment of immunosuppressive cells.^52^ Among the 16 immune cells showing significant differences, neutrophils were significantly lower in the MM samples than that in the control samples. Neutrophils are associated with antitumour functions, including cytotoxic effects on carcinoma cells and tumour growth inhibition.^53^, ^54^ In this context, *MPO*‐derived hypochlorous acid may possess anti‐oncogenic potential owing to its ability to induce tumour cell death.^55^, ^56^ *MPO* exhibits significant interactions with infiltrating immune cells in MM, such as neutrophils, suggesting its potential role in modulating tumour immunity. Hence, it is plausible that *MPO* is involved in the development and progression of MM through immune‐related pathways, emphasizing the significance of immune dysregulation in this disease. Understanding these mechanisms holds promise for the development of targeted therapies and improving patient outcomes in MM.

Currently, MR studies are gaining popularity because alleles are randomly assigned during gamete formation, making MR similar to a ‘natural’ randomised controlled trial that effectively reduces the impact of confounding and reverse causality biases.^57^, ^58^ Although various MR studies have examined the influence of risk factors on MM, they vary in design, cohort, genetic instruments and quality, leading to different estimates of causal effects. This study represents the first exploration of the causal relationship between gene levels and MM risk using a combination of microarray and MR analyses based on GEO and GWAS datasets. Our findings using the IVW method demonstrate a significant association between *MPO* and MM risk. Additionally, other methods produced similar risk estimates with consistent OR values below 1, suggesting that higher exposure is linked to reduced disease risk. The MR results aligned with previous findings that *MPO* showed a statistically significant downregulation in patients with MM. The integration of microarray analysis with the MR approach enhanced the robustness of the association between *MPO* and MM.

In this study, *MPO* was successfully identified and validated as a promising biomarker for MM through a comprehensive approach involving microarray analysis and MR. Our study identified five hub genes associated with MM based on microarray data analysis. MR corroborated the causal relationship between *MPO* levels and MM risk, thereby reinforcing the robustness of our findings. Moreover, correlation analysis revealed significant interactions between *MPO* and immune cells, suggesting that *MPO* is associated with MM cell tumorigenesis and survival through immune‐related pathways by influencing the immune cells. However, it is important to acknowledge the limitations of this study. First, although we endeavoured to minimize genetic pleiotropy, complete elimination might not have been feasible. Additionally, the biomarker may only be causative during specific life stages, potentially leading to the omission of certain causal associations. Furthermore, the modest sample sizes of our validation datasets may have compromised the reliability of the validation. Also, we only used multiple datasets from publicly available databases, which requires prospective studies to determine the efficacy of biomarkers when applied to clinical samples. Finally, most existing data are confined to the detection of changes in gene expression and require validation through additional functional experiments. Addressing these issues will be the primary focus of future research.

## 5 CONCLUSION

In our study, the combined utilization of microarray analysis and MR successfully led to the identification and validation of *MPO* as a dependent biomarker associated with MM. Functional investigations suggest a potential association between *MPO* and the tumorigenesis and survival of patients with MM through immune‐related pathways. This discovery offers valuable insights into the molecular mechanisms underlying the disease and has significant implications for the development of personalised MM treatment strategies.

## AUTHOR CONTRIBUTIONS

**Yidong Zhu:** Conceptualization (lead); writing—original draft (lead); formal analysis (lead). **Jun Liu:** Funding acquisition (equal); writing—review and editing (equal). **Bo Wang:** Funding acquisition (equal); writing—review and editing (equal).

## FUNDING INFORMATION

This work was sponsored by the National Facility for Translational Medicine (Shanghai) (TMSK‐2021‐413) and Shanghai Municipal Health Commission (202340004).

## CONFLICT OF INTEREST STATEMENT

The authors declare that they have no competing interest.

## Supporting information


Tables S1–S5.

## Data Availability

The datasets used and/or analysed during the current study are available from the corresponding author on reasonable request.
